# Different Training Durations and Frequencies of Tai Chi for Bone Mineral Density Improvement: A Systematic Review and Meta-Analysis

**DOI:** 10.1155/2021/6665642

**Published:** 2021-03-15

**Authors:** Yi Zhou, Zhi-Hui Zhao, Xiao-Hong Fan, Wei-Hong Li, Zhen Chen

**Affiliations:** ^1^Hospital of Chengdu University of Traditional Chinese Medicine, Chengdu, Sichuan 610072, China; ^2^Basic Medical College, Chengdu University of Traditional Chinese Medicine, Chengdu, Sichuan 611137, China

## Abstract

**Objective:**

Tai Chi shows potential as a safe and cost-effective intervention to improve bone mineral density (BMD). However, the various effects caused by different training durations and frequencies have not been evaluated. This updated systematic review aims to explore the effectiveness of Tai Chi in attenuating bone mineral density loss based on different training durations and frequencies.

**Methods:**

We conducted an extensive database search in Cochrane Central Register of Controlled Trials, PubMed, EMBASE, Web of Science, Chinese Biomedical Literature Database, China Knowledge Resource Integrated Database, Wanfang Data, and China Science and Technology Journal Database on randomized controlled trials that examined Tai Chi for BMD improvement. Two reviewers independently performed data screening and extraction. Study quality was evaluated using the Cochrane Handbook for Systematic Reviews of Interventions.

**Results:**

A total of 23 randomized controlled trials involving 1582 patients were identified. The aggregated results have shown significant benefits in favor of Tai Chi on BMD improvement in the lumbar spine (SMD = 0.36, 95% [0.13, 0.59], *P*=0.002), femoral neck (SMD = 0.40, 95% [0.16, 0.63], *P*=0.0009), femoral trochanter (SMD = 0.43, 95% CI [0.20, 0.66], *P*=0.0002), and Ward's triangle (SMD = 0.31, 95% [0.15, 0.48], *P*=0.002). Such favorable benefits in Tai Chi can only be seen when compared with the nonexercise group, and Tai Chi showed no significant improvement in BMD change when compared with other exercises group. Subgroup analyses showed various effects of BMD improvement based on different training durations and frequencies of Tai Chi. Tai Chi is effective in attenuating BMD loss with an intervention frequency of >4 days/week in the lumbar spine, with an intervention frequency of >4 days/week or an intervention duration of >10 months in the femoral neck, and with an intervention duration of >10 months or a frequency of ≤4 days/week in Ward's triangle.

**Conclusions:**

The results demonstrated that Tai Chi may have benefits in attenuating BMD loss. Different training durations and frequencies may result in variable effectiveness. Researchers should focus more on the training durations and frequencies of Tai Chi so that a more definitive claim can be made regarding the beneficial effects for BMD improvement.

## 1. Introduction

The decrease in bone mineral density (BMD) is closely related to age [1]. Osteoporosis caused by age-related bone loss puts a heavy burden on families and society [2, 3]. Currently, there is no safe and effective radical cure to restore osteoporotic bones, making early prevention of osteoporosis particularly important [4, 5]. The essential elements in preventing osteoporosis are achieving normal peak bone mass and attenuating age-related bone mass reduction [6]. In recent years, in addition to pharmaceutical treatments, the positive effect of exercise on bone density has attracted more and more attention [7, 8]. Studies have shown that exercise can prevent and treat osteoporosis in the following ways: (1) it can stimulate osteoblasts and bone marrow stem cells to produce biological effects through mechanical stress [9, 10], (2) it can up-regulate the expression of key factors in signaling pathways including WNT, BMP, OPG/RANK/RANKL [11, 12], and (3) it can regulate the endocrine system and increase the estrogen level in the body [13, 14]. At present, the positive effect of exercise on osteoporosis has become a research hotspot.

Tai Chi is a mind-body, low-impact, weight-bearing exercise that is growing in popularity worldwide and shows potential as a safe and cost-effective intervention to improve BMD. It is an enjoyable and gentle activity that involves the entire body with a high adherence [15]. A handful of studies have evaluated the effectiveness of Tai Chi on BMD [16–19], and more and more systematic reviews (SRs) in this field are emerging [20–23]. However, the retrieval time of the most recent SR is until 2017, and new randomized controlled trials (RCTs) have been published in the past two years. Besides, no SR focused on the BMD change in different body parts, as well as different BMD improvements caused by various intervention durations and frequencies of Tai Chi. Therefore, we conducted an updated SR to comprehensively and systematically evaluate the effects of Tai Chi on BMD change based on different training durations and frequencies and provided evidence-based recommendations to clinicians.

## 2. Methods

### 2.1. Data Sources and Search Strategies

The following databases were searched using a computer: Cochrane Central Register of Controlled Trials (CENTRAL), PubMed, EMBASE, Web of Science, Chinese Biomedical Literature Database (CBM), China Knowledge Resource Integrated Database (CNKI), Wanfang Data, and China Science and Technology Journal Database (VIP). The primary search terms used were “Taiji,” “Bone mineral density,” “Bone health,” “Bone metabolism,” “Osteoporosis.” The retrieval time was from the establishment of the database to May 2020, and only studies published in English or Chinese were included. Relevant systematic reviews and the references to the included articles were also searched to supplement other potentially relevant literature.

### 2.2. Criteria for Considering Studies for This Review

Studies should meet the following inclusion criteria (PICO format): (1) Participants: individuals without serious complications. The age, gender, case source, nationality, disease duration, or race of subjects was not restricted. (2) Interventions: the experimental group was given Tai Chi Chuan, and the style, the intervention duration, and the intervention frequency were not restricted. Other Tai Chi exercises like Tai Chi push hands or Tai Chi ball were excluded. (3) Control: any type of control group, including usual care, conventional Western medicine, no exercise, and any kind of exercise, was acceptable. (4) Outcomes: the primary outcomes were dual-energy X-ray absorptiometry measures of BMD of the spine, the femoral neck, the femoral tuberosity, and Ward's triangle. (5) Study type: RCTs.

### 2.3. Literature Screening

Two investigators reviewed the titles and abstracts independently according to the preset inclusion criteria and excluded unrelated literature. Then full-text screening of the remained studies was carried out, and the two investigators identified the final included research independently according to the inclusion criteria. The results were cross-checked, and the investigators' differences were resolved by consensus with a third investigator.

### 2.4. Data Extraction

Two reviewers extracted the data while blinded to each other's review according to the prepiloted, standardized forms. The original author(s) was contacted in case of incomplete information provided in the article. The data extraction included the following aspects: (1) general information: first author, publication year, literature topics; (2) research characteristics: baseline comparability, sample size, sex ratio, country, intervention measures, treatment course, follow-up; (3) outcome indicators; and (4) relevant factors for evaluating the risk of bias. A third reviewer was consulted if there was disagreement during cross-correction.

### 2.5. Assessment of Risk of Bias in Included Studies

Two reviewers independently assessed the risk of bias in accordance with the assessment tool suggested in the Cochrane Handbook for Systematic Reviews of Interventions [24] and then conducted cross-checking. The following aspects were included: random sequence generation (selection bias); allocation concealment (selection bias); blinding of participants and personnel (performance bias); blinding of outcome assessment (detection bias); incomplete outcome data (attrition bias); selective reporting (reporting bias); and other sources of bias [24]. Any disagreement during this procedure was resolved by consensus with a third investigator. RevMan 5.3 provided by the Cochrane Collaboration was used to created plots demonstrating the risks of bias.

### 2.6. Statistical Analysis

Revman 5.3 was also used for statistical analysis. All outcomes were continuous variables and were calculated as standard mean difference (SMD) and 95% confidence interval (CI). We extracted the mean and standard deviation of the change from baseline and transformed it into a standard format to make sure that it was implemented successfully in our analysis. The chi-square test and *I*^2^ statistic were used to check the heterogeneity of the results. *P* ≥ 0.1 or *I*^2^ < 50% was considered to indicate low heterogeneity, and a fixed-effects model was established for statistical analysis; otherwise, subgroup analyses according to control type, intervention duration, and intervention frequency were performed, as well as sensitivity analyses if necessary. Publication bias was estimated with a funnel plot.

## 3. Results

### 3.1. Literature Screening

**Figure 1:**
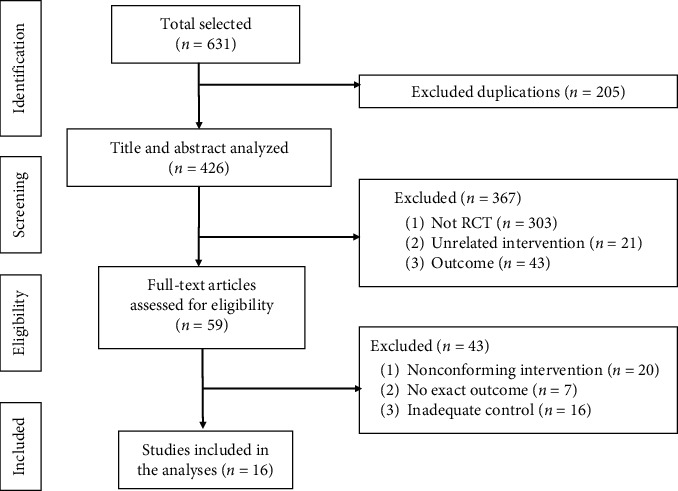
Flow chart of selection process.

Six hundred thirty-one original studies were collected by database searching, and 205 duplicate studies were excluded. After screening the titles and abstracts of the remaining literature, 367 articles were excluded, including non-RCTs and irrelevant publications. Then the full texts were read, and another 43 studies were excluded due to their nonconforming interventions, inadequate control groups, and inaccurate evaluation indicators. At last, 16 studies, 23 RCTs were included [16–18, 25–37]. The specific screening process and results are displayed in Figure 1.

### 3.2. Characteristics of Included Studies

A total of 16 studies [16–18, 25–37], 23 RCTs, involving 1582 patients were included (Table 1). The settings of the included trials were diverse and included China, South Korea [17], and the United States of America [16]. Among these 23 trials, ten trials [16, 17, 25–29, 34] had an intervention duration of ≤10 months, and 13 trials had >10 months. The intervention frequency in each RCT was also different, 10 [27, 28, 33–35, 37, 38] of which were ≤4 days/week and 13 were >4 days/week. The interventions in the control groups included no exercise [16–18, 25–28, 31–36] and other exercises such as rapid walk [30], dance [30], rope skipping [29], and resistance exercise [37]. The outcomes included were as follows: the BMD change in the spine was used in 22 trials [16, 18, 25–37], the BMD change in the femoral neck was used in 15 trials [16–18, 25–28, 30–33, 35, 36], the BMD change in the femoral tuberosity was used in 13 trials [18, 25, 30–33, 35, 36], and the BMD change in Ward's triangle was used in 12 trials [27, 30–33, 35, 36].

### 3.3. Assess of Risk of Bias

**Figure 2:**
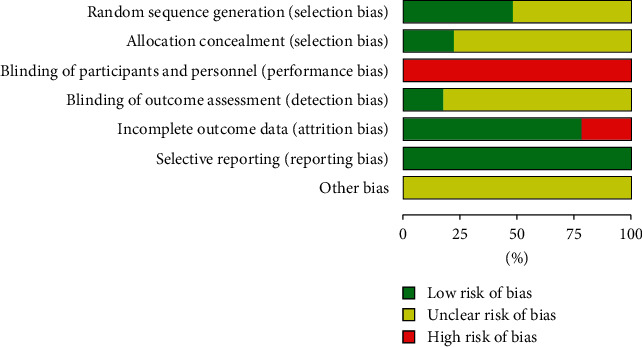
Risk of bias graph.

**Figure 3:**
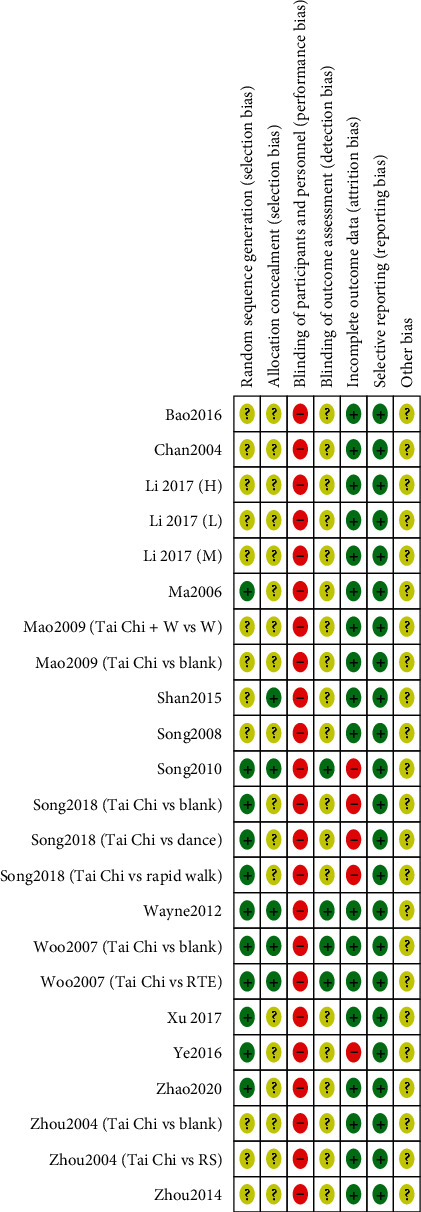
Risk of bias summary.

The risk of bias assessment of all included studies is shown in Figures 2 and 3. Four trials reported the method of randomization in terms of a computer-generated random sequence [16, 17, 37]; 6 trials used a random number table [30, 32, 35, 36]; 1 trial applied the method of drawing lots [27]; the remaining trials lacked descriptions of particular random sequence generation method. Four trials used sealed opaque envelopes to perform the allocation concealment [16, 26, 37], while the remaining trails failed to provide sufficient details about allocation concealment. Due to the apparent difference, whether to use Tai Chi or not, between the two groups, a blind method could not be used in the participants or administrators in these studies. Four trials were shown to blind its outcome assessment [16, 17, 37]. Five trials reported a high but nonrandom drop-out rate [17, 27, 30]. All studies reported all outcomes listed in their methods section. The data necessary for judging the risk of other biases in all trials were insufficient.

### 3.4. Meta-Analysis of Measured Outcomes

#### 3.4.1. BMD Change in the Lumbar spine (L2–L4)

**Figure 4:**
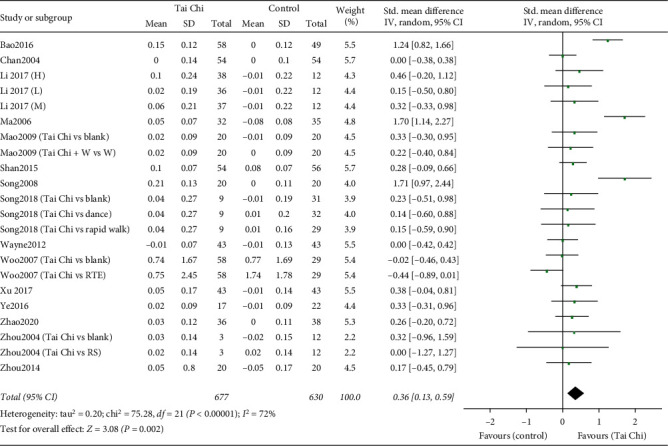
Forest plot of the comparison between the Tai Chi and the control group for the outcome BMD change in the lumbar spine (L2–L4).

A total of 22 RCTs with 1367 patients were included [16, 18, 25–37], 677 of which underwent a Tai Chi intervention. A random effect model was used to perform the meta-analysis on account of the high heterogeneity (*I*^2^ = 72%, *P* < 0.00001). The pooled result indicated a favorable effect of Tai Chi on BMD change in the spine compared to the control group (SMD = 0.36, 95% [0.13, 0.59], *P*=0.002) (Figure 4).

*Subgroup Analyses Based on Control Types, Intervention Durations, and Intervention Frequencies*. First, subgroup analysis according to control type (nonexercise and other exercises) was performed. Four trials [29, 30, 37] reported a control type of other exercises, while 18 trials [16, 18, 25–37] reported no exercise. The pooled result showed that Tai Chi did not significantly improve BMD of the lumbar spine (SMD = −0.18, 95% [−0.51, 0.15], *P*=0.29) compared to other exercises group with low heterogeneity (*I*^2^ = 0%, *P*=0.43), and that Tai Chi was superior to nonexercise group in improving BMD of the lumbar spine (SMD = 0.44, 95% [0.19, 0.68], *P*=0.0005) with high heterogeneity (*I*^2^ = 73%, *P* < 0.00001).

Then, we conducted subgroup analyses according to intervention durations (>10 months and ≤10 months) and intervention frequencies (>4 days/week and ≤4 days/week) in the nonexercise group (Table 2). The pooled results showed that Tai Chi significantly improved BMD of the lumbar spine with an intervention frequency of >4 days/week (SMD = 0.67, 95% [0.25, 1.09], *P*=0.002) [18, 25, 26, 28, 30–33, 36] rather than of ≤4 days/week (SMD = 0.16, 95% [−0.02, 0.35], *P*=0.09) [16, 27, 29, 33–35, 37], and that Taiji was superior to no exercise group in improving BMD of the lumbar spine with intervention durations of either >10 months (SMD = 0.50, 95% [0.13, 0.86], *P*=0.008) [18, 30–33, 35–37] or ≤10 months (SMD = 0.38, 95% [0.04, 0.71], *P*=0.03) [25–29, 34].

#### 3.4.2. BMD Change in the Femoral Neck

**Figure 5:**
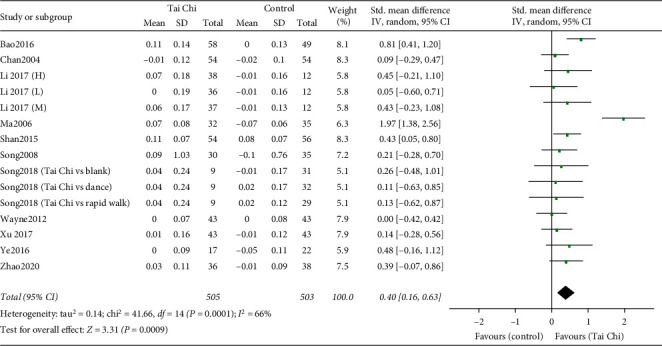
Forest plot of the comparison between the Tai Chi and the control group for the outcome BMD change in the femoral neck.

Fifteen RCTs were included with a total of 1008 patients [16, 18, 25–27, 30–33, 35, 36]. The pooled result showed a significant difference between the two groups in BMD change in the femoral neck (SMD = 0.40, 95% [0.16, 0.63], *P*=0.0009), with high heterogeneity (*I*^2^ = 66%, *P*=0.0001) (Figure 5).

*Subgroup Analyses Based on Control Types, Intervention Durations, and Intervention Frequencies*. We firstly conducted subgroup analysis according to control type (nonexercise and other exercises). For the comparison between the Tai Chi and the other exercises group, the pooled data from 2 trials [30] showed that no significant difference was found in BMD improvement in the femoral neck (SMD = 0.12, 95% [−0.41, 0.64], *P*=0.67) between the two groups with low heterogeneity (*I*^2^ = 0%, *P*=0.97). For the comparison between the Tai Chi and the nonexercise group, the combined result of 18 RCTs [16, 18, 25–27, 30–33, 35, 36] showed that Tai Chi could significantly improve BMD of the femoral neck (SMD = 0.43, 95% [0.17, 0.68], *P*=0.001) with high heterogeneity (*I*^2^ = 70%, *P* < 0.0001).

Subgroup analyses according to intervention durations (>10 months and ≤10 months) and intervention frequencies (>4 days/week and ≤4 days/week) were secondly carried out in the nonexercise group (Table 3). The pooled results showed that Tai Chi had a better effect on BMD improvement with an intervention frequency of >4 days/week (SMD = 0.53, 95% [0.15, 0.91], *P*=0.006) [18, 25, 26, 30–32, 34, 36] and an intervention duration of >10 months (SMD = 0.51, 95% [0.12, 0.91], *P*=0.01) [26, 30, 32, 33, 35, 36]. No significant difference between the Tai Chi and the no exercise group in BMD improvement of the femoral neck was found in an intervention duration of ≤10 months (SMD = 0.31, 95% [−0.01, 0.63], *P*=0.06) [16, 18, 25, 27, 31] and an intervention frequency of ≤4 days/week (SMD = 0.24, 95% [0.00, 0.48], *P*=0.05) [16, 27, 33, 35].

#### 3.4.3. BMD Changes in the Femoral Trochanter

**Figure 6:**
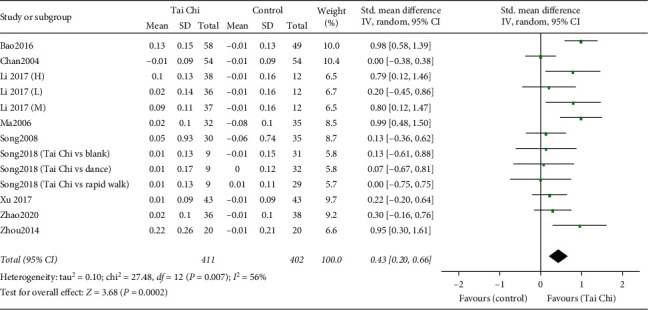
Forest plot of the comparison between the Tai Chi and the control group for the outcome BMD change in the femoral trochanter.

Thirteen trials involving 813 participants examined changes in the femoral trochanter [18, 25, 28, 30–33, 35, 36]. The combined result showed a significant difference between the two groups (SMD = 0.43, 95% CI [0.20, 0.66], *P*=0.0002) with high heterogeneity (*I*^2^ = 56%, *P*=0.007) (Figure 6).

*Subgroup Analyses Based on Control Types, Intervention Durations, and Intervention Frequencies.* Subgroup analysis according to control type (nonexercise and other exercises) was first conducted. Pooled BMD changes in the femoral trochanter improved significantly in the Tai Chi group compared to the nonexercise group (SMD = 0.49, 95% [0.23, 0.74], *P*=0.0002) with high heterogeneity (*I*^2^ = 60%, *P*=0.005) [18, 25, 28, 30–33, 35, 36]. No significant difference between the Tai Chi and the other exercises group was found (SMD = 0.04, 95% [−0.49, 0.56], *P*=0.89) with low heterogeneity (*I*^2^ = 70%, *P* < 0.0001) [30].

We subsequently conducted subgroup analyses according to intervention durations (>10 months and ≤10 months) and intervention frequencies (>4 days/week and ≤4 days/week) in the nonexercise group (Table 4). The combined results showed that Tai Chi was superior to the nonexercise group with either an intervention duration of ≤10 months (SMD = 0.68, 95% [0.11, 1.26], *P*=0.02) [25, 28, 31] or a duration of >10 months (SMD = 0.41, 95% [0.15, 0.67], *P*=0.002) [18, 30, 32, 33, 35, 36], as well as with either an intervention frequency of ≤4 days/week (SMD = 0.52, 95% [0.17, 0.87], *P*=0.003) [28, 33, 35] or a frequency of >4 days/week (SMD = 0.46, 95% [0.11, 0.81], *P*=0.009) [18, 25, 30–33, 36].

#### 3.4.4. BMD Changes in Ward's Triangle

**Figure 7:**
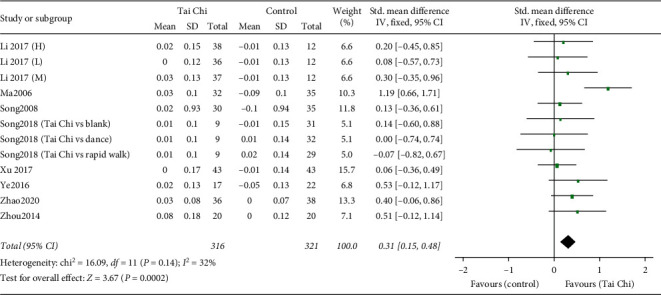
Forest plot of the comparison between the Tai Chi and the control group for the outcome BMD change in Ward's triangle.

Twelve RCTs reported BMD changes in Ward's triangle, with 637 individuals [27, 28, 30–33, 35, 36]. The 12 trials' heterogeneity was relatively low (*I*^2^ = 32%, *P*=0.14), so we chose to conduct a quantitative synthesis using a fixed effect model. The combined result was statistically significant (SMD = 0.31, 95% [0.15, 0.48], *P*=0.002) compared to the control group, showing favorable effects of Taiji on BMD changes in Ward's triangle (Figure 7).

*Subgroup Analyses Based on Control Types, Intervention Durations, and Intervention Frequencies.* Firstly, we carried out a subgroup analysis according to the control type (nonexercise and other exercises). The pooled result of 10 studies comparing the Tai Chi and the nonexercise group showed a significant difference (SMD = 0.36, 95% [0.13, 0.58], *P*=0.002) with low heterogeneity (*I*^2^ = 37%, *P*=0.12) [27, 28, 30–33, 35, 36], while no significant difference was found between the Tai Chi and other exercises group (SMD = −0.04, 95% [−0.56, 0.49], *P*=0.89) with low heterogeneity (*I*^2^ = 0%, *P*=0.89) [30].

Secondly, subgroup analyses according to intervention durations (>10 months and ≤10 months) and intervention frequencies (>4 days/week and ≤4 days/week) were carried out in the nonexercise group (Table 5). The combined results showed that Tai Chi was superior to the nonexercise group with an intervention duration of >10 months (SMD = 0.38, 95% [0.03, 0.73], *P*=0.03) [32, 33, 35, 36] rather than of ≤10 months (SMD = 0.31, 95% [0.00, 0.61], *P*=0.05) [27, 28, 30], as well as with an intervention frequencies of ≤4 days/week (SMD = 0.37, 95% [0.11, 0.64], *P*=0.006) [27, 28, 33, 35] rather than of >4 days/week (SMD = 0.35, 95% [−0.09, 0.79], *P*=0.12) [30–33, 36].

#### 3.4.5. Publication Bias

**Figure 8:**
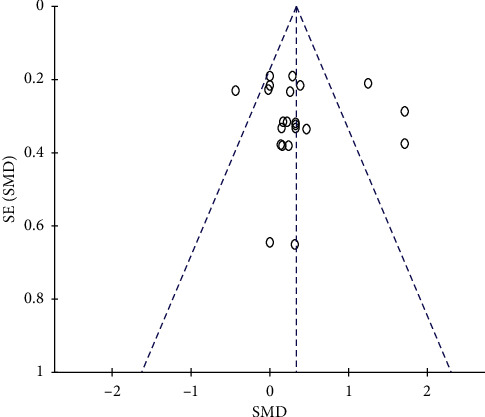
Evaluation of publication bias for outcome of BMD change in the lumbar spine (L2–L4).

The funnel plot was drawn based on RCTs that included the outcome of BMD change in the lumbar spine (L2–L4). The funnel plot was obviously asymmetrical, suggesting that publication bias might exist (Figure 8). Four RCTs, which were significantly a skewed in the graph, showed no difference in methodology and other aspects [25, 31, 36, 37].

#### 3.4.6. Safety Monitoring

Three RCTs reported that there were no serious adverse events [16, 35, 36], while the remaining trials did not mention adverse events.

## 4. Discussion

Currently, there are several meta-analyses published on the same topic, as presented in Table 6. The highlights of our work are as follows: (1) firstly, our analyses included new RCTs that were published in the past two years. Since the results of previous systematic reviews were not uniform, the inclusion of further RCTs can lead to more accurate conclusions; (2) secondly, we added some results, including BMD change in the femoral trochanter and Ward's triangle, to study the effect of Tai Chi on different parts of the body; (3) lastly, we conducted subgroup analyses on control type, intervention duration, and intervention frequency. In this way, the effects caused by different training durations and frequencies were evaluated, and we found that different training durations and frequencies of Tai Chi could result in variable effectiveness.

### 4.1. Main Results and Analysis

This systematic review and meta-analysis, which was based on 23 RCTs involving 1582 participants, found that Tai Chi may have a positive effect on BMD improvement in the lumbar spine, the femoral neck, the femoral trochanter, and Ward's triangle. Our study showed benefits in improving BMD in the four parts mentioned above in favor of Tai Chi compared with the nonexercise group. However, no significant improvement was found between the Tai Chi and the other exercises group. Besides, different intervention durations and frequencies of Tai Chi may lead to various effects. For BMD in the lumbar spine, only Tai Chi with an intervention frequency of >4 days/week was shown to have a beneficial effect, while no significant improvement was found with an intervention frequency of ≤4 days/week. For BMD in the femoral neck, the pooled result showed that significant improvement could only be found in subgroups of an intervention frequency of >4 days/week and an intervention duration of >10 months. For BMD in the femoral trochanter, Tai Chi was shown to have a beneficial effect in all durations (≤10 months and >10 months) and frequencies (≤4 days/week and >4 days/week). For BMD in Ward's triangle, a beneficial effect of Tai Chi was found only after an intervention duration of >10 months or a frequency of ≤4 days/week.

Tai Chi is a moderate-intensity aerobic exercise, characterized by a high demand for neuromuscular coordination, low velocity of muscle contraction, and no jumping. Tai Chi affects bone health through the two following aspects: one is the mechanical regulation system, that is, the beneficial effect on bone comes from the mechanical stress generated during the Tai Chi exercise; the other is the hormone regulation system, and one study found that the serum bone-specific alkaline phosphatase (BAP) concentration and the ratio of BAP to pyridinoline (BAP/PYD) increased significantly after six weeks of Tai Chi exercise, indicating the bone-promoting effect in Tai Chi [39]. In addition, Tai Chi also plays a positive role in psychological adjustment [40]. Although showing no significant BMD improvement compared to other exercises, including resistance exercise, rapid walking, dancing, and rope skipping, Tai Chi is still recommended as a suitable exercise for older patients with rheumatoid arthritis and osteoporotic conditions for smooth, slow, calm, and conscious-based movements, which are barely seen in other strengthening exercises [41]. Till now, some studies have focused on the effectiveness of different training frequencies and durations in Tai Chi. One study has found that Tai Chi was shown to have a significant improvement that is positively related to the intervention duration in functional gait assessed by timed up-and-go tests [42]. Another study has found that a brief high-impact exercise every day is more beneficial to BMD improvement than an exercise of 4 days per week [43]. According to our review, different training durations and frequencies could result in variable effectiveness. However, the interpretation of these results should be treated with caution. In this meta-analysis, high heterogeneity, a relatively small number of studies, and generally low quality of included literature raised concerns about the accuracy of the conclusion. A well-designed, large-scale trial is still needed to validate this result.

### 4.2. Limitations

The overall quality of the included studies was not high. Only 11 RCTs reported appropriate randomization methods [16, 17, 27, 30, 32, 35–37], and 4 RCTs mentioned allocation concealment [16, 26, 37]. Meanwhile, performance bias could not be ruled out because participants cannot be blinded to the Tai Chi exercise. In addition, four trials mentioned blinding of outcome assessment [16, 17, 37], and 5 RCTs reported a high but nonrandom drop-out rate [17, 27, 30]. At the same time, publication bias also existed. Four RCTs were significantly asymmetrical according to the funnel plot [25,31,36,37]. However, differences in the design and methodology of these RCTs cannot be found, which may be caused by the low quality and inadequate information provided in these studies.

The heterogeneity among the studies was significant. Subgroup analyses with respect to control type, intervention duration, and intervention frequency were subsequently carried out, and we found that these three aspects might partly be the source of heterogeneity. However, the above-mentioned three categories can only explain the heterogeneity to some extent and should be interpreted with great caution. There remained substantial unexplained heterogeneity in this review, including study design and study quality. For example, the detailed included participants in each study were slightly different: some were menopausal women, some were elderly women, some were elderly men, and some had hyperlipidemia. Besides, the training style and daily practice time in each study were also different. Various training styles of Tai Chi involving Yang-style, Sun-style, and 24-form were included in this review, and the daily practice time was not the same, ranging from 30 to 90 minutes per day. All these differences may result in high heterogeneity.

The distribution of the included studies is uneven. Most studies were conducted in Asian countries, and only 1 study was from the USA. Therefore, ethnic-based subgroup analysis could not be carried out. Besides, we only performed a search for English and Chinese studies, but other languages should be searched to expand the scope. In the absence of enough data, this study only focused on the efficacy of Tai Chi on BMD improvement, but other aspects, including muscle strength, functional mobility, and fracture incidence, may also be assessed.

### 4.3. Practical Implications

We synthesized current data of Tai Chi on BMD improvement and found that Tai Chi seemed to be an effective exercise therapy to improve BMD in the spine, the femoral neck, the femoral trochanter, and Ward's triangle. The current finding, which should be interpreted with caution on account of the low methodological quality and the high heterogeneity, is still a promising reference for future clinical trials. Future studies should focus more on the effectiveness of various training types, frequencies, and durations of Tai Chi, and create a set of standard Tai Chi exercises with specific moves, practice frequency, and training duration for global promotion.

## 5. Conclusions

According to our review, Tai Chi may be effective for BMD improvement and can be promoted as cost-effective exercise therapy. The training time and frequency required for various parts are different. However, due to the limitations of the included studies, large-sample, multi-center, well-designed clinical trials are still required to verify this conclusion. Moreover, future studies should focus more on the relationship between the training time and frequency of Tai Chi and different body parts to create a set of standard Tai Chi exercises with specific moves, practice frequency, and training duration.

## Figures and Tables

**Table 1:** Characteristics of included studies.

Author year	Location	Participants	No. (T/C)	Tai Chai group			Control group	Training duration (months)	Outcome
				Style or form	Daily time	Frequency (days/week)			
Xu, 2017	China	Menopausal women	43/43	24 form	≥40 min	6	Nonexercise	12	①②③④
Li, 2017 (L)^*∗*^	China	Women aged 60 to 70	36/12	24 form	60 min	1	Nonexercise	12	①②③④
Li, 2017 (M)^*∗*^	China	Women aged 60 to 70	37/12	24 form	60 min	3	Nonexercise	12	①②③④
Li, 2017 (M)^*∗*^	China	Women aged 60 to 70	38/12	24 form	60 min	6	Nonexercise	12	①②③④
Song, 2018^#^	China	Women aged 60 to 70	9/29	24 form	70 min	5	Rapid working	12	①②③④
Song, 2018^#^	China	Women aged 60 to 70	9/32	24 form	70 min	5	Dancing	12	①②③④
Song, 2018^#^	China	Women aged 60 to 70	9/31	24 form	70 min	5	Nonexercise	12	①②③④
Song, 2008	China	Patients with primary osteoporosis	20/20	24 form	60 min	6	Nonexercise	12	①②
Mao, 2009^§^	China	Retired women	20/20	Not reported	30 min	3	Nonexercise	5	①
Mao, 200^§^	China	Retired women	20/20	Not reported	30 min	3	Western medicine	5	①
Zhou, 2014	China	Patient with hyperlipidemia	20/20	Not reported	90 min	4	Nonexercise	6	①②③④
Ye, 2016	China	Middle-aged and elderly women	25/25	Not reported	30–60 min	3	Nonexercise	6	①②④
Shan, 2015	China	Menopausal women	60/60	24 form	60 min	7	Nonexercise	6	②
Ma, 2006	China	Male	32/35	24 form	60 min	7	Nonexercise	12	①②③④
Bao, 2016	China	Patients with type 2 diabetes	58/49	Not reported	4 hours	7	Nonexercise	6	①③④
Zhao, 2020	China	Menopausal women	36/38	24 form	60 min	3	Nonexercise	12	①②③④
Chan, 2004	China	Menopausal women	67/65	Yang-style	45 min	5	Nonexercise	12	①③
Woo, 2007^^^	China	Participants aged 65 to 74	58/29	Yang-style	Not reported	3	Resistance exercise	12	①
Woo, 2007^^^	South Korea	Participants aged 65 to 74	58/29	Yang-style	Not reported	3	Nonexercise	12	①
Song, 2010	South Korea	Women with osteoarthritis	41/41	Sun-style	45–60 min	7	Nonexercise	6	②③④
Wayne, 2012	USA	Post-menopausal osteoarthritis women	43/43	Not reported	30–60 min	4	Nonexercise	9	①
Zhou, 2004^£^	China	Menopausal women	3/12	24 or 42 form	45–60 min	7	Nonexercise	10	①
Zhou, 2004^£^	China	Menopausal women	3/12	24 or 42 form	45–60 min	7	Rope skiing	10	①

① BMD in the lumbar spine; ② BMD in the femoral neck; ③ BMD in the femoral trochanter; ④ BMD in Ward's triangle;^*∗*^^#^£^ RCTs with the same superscript belong to one study. T/C: Tai Chi/Control.

**Table 2:** Subgroup analyses between the Tai Chi and the nonexercise group for the outcome BMD change in the lumbar spine (L2–L4) according to intervention durations and frequencies.

Subgroup	*n*	SMD, 95% CI	*P*	Heterogeneity
Intervention duration				
≤10 months	8	0.38, 95% [0.04, 0.71]	0.03	*I* ^2^ = 66%, *P* = 0.005
>10 months	10	0.50, 95% [0.13, 0.86]	0.008	*I* ^2^ = 78%, *P* < 0.00001

Intervention frequencies				
≤4 days/week	9	0.16, 95% [−0.02, 0.35]	0.09	*I* ^2^ = 0%, *P* = 0.97
>days/week	9	0.67, 95% [0.25, 1.09]	0.002	*I* ^2^ = 84%, *P* < 0.00001

**Table 3:** Subgroup analyses between the Tai Chi and the nonexercise group for the outcome BMD change in the femoral neck according to intervention durations and frequencies.

Subgroup	*n*	SMD, 95% CI	*P*	Heterogeneity
Intervention duration				
≤10 months	5	0.31, 95% [−0.01, 0.63]	0.06	*I* ^2^ = 59%, *P* = 0.04
>10 months	8	0.51, 95% [0.12, 0.91]	0.01	*I* ^2^ = 76%, *P* = 0.0001

Intervention frequencies				
≤4 days/week	5	0.24, 95% [−0.00, 0.48]	0.05	*I* ^2^ = 0%, *P* = 0.59
>days/week	8	0.53, 95% [0.15, 0.91]	0.006	*I* ^2^ = 80%, *P* < 0.00001

**Table 4:** Subgroup analyses between the Tai Chi and the nonexercise group for the outcome BMD change in the femoral trochanter according to intervention durations and frequencies.

Subgroup	*n*	SMD, 95%CI	*P*	Heterogeneity
Intervention duration				
≤10 months	3	068, 95% [0.11, 1.26]	0.02	*I* ^2^ = 74%, *P* = 0.2
>10 months	8	0.41, 95% [0.15, 0.67]	0.002	*I* ^2^ = 51%, *P* = 0.05

Intervention frequencies				
≤4 days/week	4	0.52, 95% [0.17, 0.87]	0.003	*I* ^2^ = 27%, *P* = 0.25
>days/week	7	0.46, 95% [0.11, 0.81]	0.009	*I* ^2^ = 80%, *P* < 0.002

**Table 5:** Subgroup analyses between the Tai Chi and the nonexercise group for the outcome BMD change in Ward's triangle according to intervention durations and frequencies.

Subgroup	*n*	SMD, 95% CI	*P*	Heterogeneity
Intervention duration				
≤10 months	4	0.31, 95% [0.00, 0.61]	0.05	*I* ^2^ = 0%, *P* = 0.66
>10 months	6	0.38, 95% [0.03, 0.73]	0.03	*I* ^2^ = 60%, *P* = 0.03

Intervention frequencies				
≤4 days/week	5	0.37, 95% [0.11, 0.64]	0.006	*I* ^2^ = 0%, *P* = 0.87
>days/week	5	0.35, 95% [−0.09, 0.79]	0.12	*I* ^2^ = 69%, *P* = 0.01

**Table 6:** Comparisons with other previous meta-analysis.

Author, year	Sun 2016	Liu 2017	Zou 2017	Zhang 2019	The present meta-analysis
Number of RCTs	11	6	20	15	23
Participants	Perimenopausal and postmenopausal women	Postmenopausal women	Middle-aged and older adults	Osteopenia and primary osteoporosis	Individual without serious diseases
Search strategy until (year)	2015	2016	2016	2017	20
Subgroup analysis	Control type	NA	NA	Control type, participants	Control type, intervention duration, and intervention frequency
Outcomes	BMD in the lumbar spine	BMD in the lumbar spine and the femoral neck	BMD in the lumbar spine, the femoral neck, and the femoral trochanter.	BMD	BMD in the lumbar spine, the femoral neck, the femoral tochanter, and Ward's triangle

## Data Availability

All data generated or analyzed during this study are included within this article.

## Abbreviations

BMD: Bone mineral density
SR: Systematic review
RCT: Randomized controlled trial
CENTRAL: Cochrane Central Register of Controlled Trials
CBM: Chinese Biomedical Literature Database
CNKI: China Knowledge Resource Integrated Database
VIP: China science and technology journal database
SMD: Standard mean difference
CI: Confidence interval.
